# Lipoplexes to Deliver Oligonucleotides in Gram-Positive and Gram-Negative Bacteria: Towards Treatment of Blood Infections

**DOI:** 10.3390/pharmaceutics13070989

**Published:** 2021-06-29

**Authors:** Sara Pereira, Rita Sobral Santos, Luís Moreira, Nuno Guimarães, Mariana Gomes, Heyang Zhang, Katrien Remaut, Kevin Braeckmans, Stefaan De Smedt, Nuno Filipe Azevedo

**Affiliations:** 1 Laboratory for Process Engineering, Environment, Biotechnology and Energy (LEPABE), Faculty of Engineering, University of Porto, 4200-465 Porto, Portugal; up201610825@fe.up.pt (S.P.); luismoreira@fe.up.pt (L.M.); nguimaraes@fe.up.pt (N.G.); mggomes@fe.up.pt (M.G.); nazevedo@fe.up.pt (N.F.A.); 2 Ghent Research Group on Nanomedicine, Laboratory of General Biochemistry and Physical Pharmacy, Faculty of Pharmaceutical Sciences, Ghent University, 9000 Ghent, Belgium; Heyang.Zhang@ugent.be (H.Z.); Katrien.Remaut@ugent.be (K.R.); Kevin.Braeckmans@ugent.be (K.B.); Stefaan.Desmedt@ugent.be (S.D.S.); 3 Centre for Advanced Light Microscopy, Ghent University, 9000 Ghent, Belgium

**Keywords:** bacteria, bloodstream infections, liposomes, locked nucleic acid, oligonucleotides

## Abstract

Bacterial resistance to antibiotics threatens the ability to treat life-threatening bloodstream infections. Oligonucleotides (ONs) composed of nucleic acid mimics (NAMs) able to inhibit essential genes can become an alternative to traditional antibiotics, as long as they are safely transported in human serum upon intravenous administration and they are carried across the multilayered bacterial envelopes, impermeable to ONs. In this study, fusogenic liposomes were considered to transport the ONs and promote their internalization in clinically relevant bacteria. Locked nucleic acids and 2′-OMethyl RNA were evaluated as model NAMs and formulated into DOTAP–DOPE liposomes followed by post-PEGylation. Our data showed a complexation stability between the post-PEGylated liposomes and the ONs of over 82%, during 24 h in native human serum, as determined by fluorescence correlation spectroscopy. Quantification by a lipid-mixing assay showed that liposomes, with and without post-PEGylation, fused with all bacteria tested. Such fusion promoted the delivery of a fraction of the ONs into the bacterial cytosol, as observed by fluorescence in situ hybridization and bacterial fractionation. In short, we demonstrated for the first time that liposomes can safely transport ONs in human serum and intracellularly deliver them in both Gram-negative and -positive bacteria, which holds promise towards the treatment of bloodstream infections.

## 1. Introduction

Antimicrobial resistance (AMR) is an increasing major public health concern, and the scientific community is struggling to find alternatives as we are entering the post-antibiotic era [1]. During the last two decades, multidrug-resistant strains have been contributing to the rate of septicemia-related hospitalizations and associated mortality [2,3]. Additionally, antimicrobial drug discovery is not keeping pace with the rates at which new multidrug-resistant strains emerge. Therefore, there is an urgent requirement for new and innovative drugs to treat bloodstream infections [4].

Oligonucleotides (ONs) composed of nucleic acid mimics (NAMs) such as locked nucleic acids (LNAs), able to resist the degradation by nucleases and with impressive binding affinity for complementary RNA, have the potential to modulate the expression of specific genes and hold promise as alternative drugs to conventional antibiotics [5,6,7]. They are easily designed to inhibit the expression of an essential bacterial gene, thus leading to bacterial cell death, or inhibit a gene associated with antibiotic resistance, thus turning the bacteria susceptible to antibiotics again [8,9,10]. In striking contrast to conventional antibiotics, this strategy provides a potentially endless source of active therapeutic agents against bacterial infections with a reduced development time. Even if the bacterial target undergoes a point mutation, the oligonucleotide can be easily redesigned to become effective again. Nevertheless, the effectiveness of ONs is very dependent on their permeation through the complex multilayered bacterial cell envelope in order to reach the intracellular space [11]. However, ONs are usually very polar, negatively charged and too large to spontaneously translocate through the bacterial envelope [12,13]. Therefore, they need the help of delivery systems to enter bacterial cells.

Liposomes are currently one of the most biocompatible and widely used carriers in the pharmaceutical industry, with several liposomal formulations already on the market targeting human cells [14,15,16]. In bacteria, they are mostly studied for the controlled release of antibiotics, but the potential of these carriers to deliver ONs into bacteria has only recently started to be studied [17,18,19,20,21]. Furthermore, their ability to deliver the cargo across different bacterial envelopes is far less understood, as they lack an endocytosis process as opposed to human cells.

To treat septicemia, the liposomes not only need to deliver the ONs across the bacterial envelope, but also need to safely carry the ONs in human blood on their way to bacteria. The association of ONs with liposomes can be severely destabilized by human serum, due to the presence of lipases, nucleases and high-density proteins [22,23]. Polyethylene glycol (PEG) is the most popular shielding polymer known to prevent serum proteins from binding to carriers, significantly improving the half-life of the formulation. On the other hand, it is reported that PEG may decrease interaction with cells, resulting in lower uptake in mammalian cells, even though this is not well characterized for bacteria [24,25,26,27].

Our group has pioneered the use of liposomes to deliver NAM-composed ONs into bacteria and already showed successful delivery by DOTAP–DOPE liposomes into *H. pylori* [19]. Being a fusogenic lipid, DOPE is expected to facilitate liposome delivery of ONs in bacteria, as it potentiates fusion with membranes [19,28]. In this study, we aimed to quantify the fusogenic characteristics of those liposomes and evaluate their ability to deliver ONs into several Gram-negative and Gram-positive bacteria. We also aimed to determine the stability of complexation between the liposomes and the ONs in native human serum via fluorescence correlation spectroscopy (FCS). A lipid-mixing assay based on fluorescence resonance energy transfer (FRET) was used to quantify the fusion of liposomes with different clinically relevant bacteria. Fluorescence in situ hybridization (FISH) and confocal microscopy were used to inspect the interaction of lipoplexes with the bacteria and internalization of ONs. Finally, a fractionation protocol was used to study the cellular localization of ONs upon delivery and to quantify their cellular distribution.

## 2. Materials and Methods

### 2.1. Materials

(2,3-Dioleoyloxy-propyl)-trimethylammonium-chloride (DOTAP), 1,2-dioleoyl-sn-glycero-3-phosphoethanolamine (DOPE), 1,2-Distearoyl-sn-glycero-3-phosphoethanolamine-*N*-(methoxy(polyethyleneglycol)-2000) (DSPE-PEG), 1,2-dioleoyl-sn-glycero-3-phosphoethanolamine-*N*-(lissamine rhodamine B sulfonyl) (Rh-PE) and 1,2-dioleoyl-sn-glycero-3-phosphoethanolamine-*N*-(7-nitro-2-1,3-benzoxadiazol-4-yl) (NBD-PE) were purchased from Avanti Polar Lipids (Alabaster, AL, USA). Chloroform, 4-(2-hydroxyethyl)-1-piperazineethanesulfonic acid (HEPES), 2-amino-2-(hydroxymethyl)-1,3-propanediol hydrochloride (Tris-HCl), sodium chloride (NaCl), Triton X-100 and 4′,6-diamidino-2-phenylindole (DAPI) were purchased from Sigma-Aldrich (Bornem, Belgium). Paraformaldehyde was purchased from Fluka (Buchs, Switzerland). Urea was acquired from VWR (Haasrode, Belgium). Tryptic soy broth (TSB) was purchased from Merck Millipore (Madrid, Spain).

### 2.2. Design of NAM-ONs

In order to study the efficiency of liposome delivery in different bacteria, a model NAM-ON that hybridizes with all eubacteria was used. As such, the ON EUB338 was chosen since it is complementary to a conserved 16S rRNA sequence present in the domain Bacteria. The ON sequence used is composed of the NAMs locked nucleic acids (LNA “+”) and 2′-OMethylRNA (2′OMe “m”) and possesses phosphorothioate (PS) internucleotide linkages (“*”): 5′+T*mG*mC*+C*mU*mC*+C*mC*mG*+T*mA*mG*+G*mA-3′. The ON was fluorescently labeled at 5′ with HiLyte 488 and was purchased from Eurogentec (Seraing, Belgium).

### 2.3. Preparation of Liposomes and Lipoplexes

**Figure 1:**
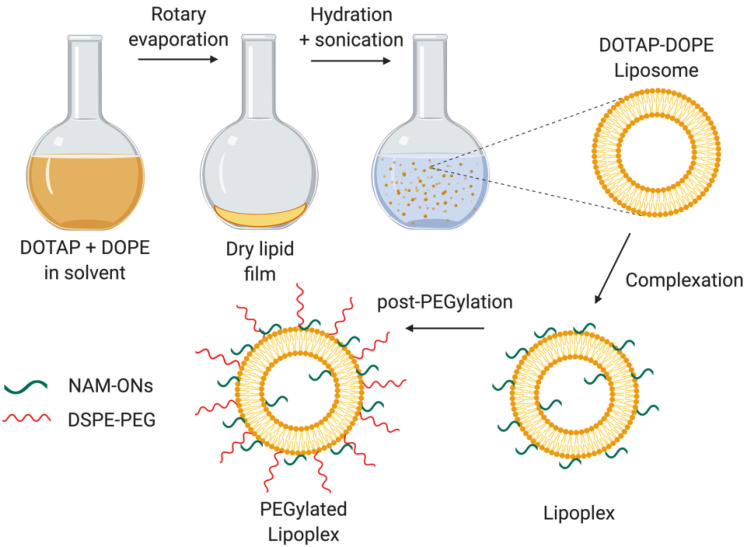
Schematic representation of the preparation of the PEGylated DOTAP–DOPE liposomes and lipoplexes. The liposomes, resulting from the hydration of a lipid film composed of DOTAP and DOPE, were incubated with the negatively charged HiLyte 488 labeled ONs to form lipoplexes. The post-PEGylation was achieved by adding DSPE-PEG at a final concentration of 10%. Created using BioRender.com. (Accessed on 1 June 2021).

DOTAP and DOPE liposomes were prepared (Figure 1) as reported before [19]. In brief, the lipids (1:1 mol ratio) were mixed in a round-bottomed flask, and a lipid film was formed by evaporation of the chloroform in a rotary evaporator programmed at 40 °C. The dried lipid film was rehydrated with 20 mM HEPES buffer (pH 7.4), resulting in a final concentration of 5 mM of each lipid. The resulted mixture was sonicated using a probe sonicator (Branson Ultrasonics Digital Sonifier, Danbury, CT, USA). The average size and zeta potential of the liposomes were routinely checked by dynamic light scattering (Zetasizer Nano-ZS, Malvern, Worcestershire, UK).

For interaction and fusion studies of the liposomes with different bacterial envelopes, empty liposomes were used. For the initial interaction assay based on epifluorescence microscopy, Rh-PE at a final concentration of 0.5% (*v*/*v*) of total lipids was added in the initial phospholipid mixture. For the fusion assays, additionally to Rh-PE, NBD-PE was also added to the initial phospholipid mixture so that the final concentration of the labeled lipids resulted in 0.5% (*v*/*v*) each. In both cases, the liposomes were diluted to 1 mM (each lipid) in HEPES.

In order to evaluate the ability of the liposomes to stably associate with NAM-ONs and deliver them in bacteria, lipoplexes were prepared by mixing the HiLyte 488 labeled ONs with the liposomes, at a ± charge ratio of 15, calculated by dividing the molar amount of positive charges on the DOTAP molecules by the molar amount of negative charges on the ON (with each NAM monomer containing 1 negatively charged phosphate group, totalizing 14 negative charges in the ON). The complexation was achieved after incubation for 30 min, at room temperature.

Post-insertion of PEG lipids (2 kDa DSPE-PEG) was performed to obtain post-PEGylated liposomes or lipoplexes [19]. Briefly, a film formed by PEG lipids was obtained by evaporation of the chloroform, via nitrogen flush, and re-dissolution in sterile milli-Q water. PEG chains were added to the lipoplexes accounting for 10% of the total lipids and the mixture was incubated for 1 h, at 37 °C (Figure 1). The efficiency of PEGylation was evaluated from the decrease in the absolute zeta potential value of the lipoplexes, measured with the Zetasizer Nano-ZS (Malvern Instruments Ltd., Worcestershire, UK)

### 2.4. Collection of Human Serum

Human serum collected from a healthy donor was obtained from the Ghent University Hospital and added to Venosafe 6 mL tubes containing gel and clotting activator (Terumo Europe, Leuven, Belgium). The tubes were centrifuged for 10 min at 4000× *g*, and the resulting serum was portioned into 500 μL aliquots and stored at −20 °C until use.

### 2.5. Determination of Complexation Stability of Lipoplexes in Buffer and Human Serum by Fluorescence Correlation Spectroscopy (FCS)

To understand if the formulation is adequate for intravenous administration, the stability of complexation between the liposomes and ONs was characterized by single-color FCS upon incubation in undiluted human serum. 

FCS is a microscopy-based technique that monitors the fluorescence fluctuations of the diffusing molecules in a focal volume of a confocal microscope. The degree of mobility of the diffusing species and their size is determined by the duration of the fluctuations. From the fluorescence fluctuations, an autocorrelation curve G(t) can be derived and the average number of molecules (N) in the focal volume and their diffusion characteristics can be calculated by the fitting of the autocorrelation curve [29,30]. The autocorrelation analysis can be useful to study fluorescently labeled molecules and their interaction with unlabeled particles, for which both populations and their diffusion coefficient can be determined. We wanted to study two different subpopulations, being one the free ONs and the other being the fraction of the ONs that is complexed with the liposomes (slower rate of diffusion). FCS measurements were performed with a photon-counting instrument (PicoHarp 300, PicoQuant, Berlin, Germany) installed on a C1si laser scanning confocal microscope (Nikon, Tokyo, Japan). The laser beam was focused through a water immersion objective lens (Plan Apo 60×, NA 1.2, collar rim collection, Nikon, Tokyo, Japan) at about 50 μm and held stationary above the glass-bottom 96-well plate (Grainer Bio-one, Frickenhausen, Germany). Each well contained 5 μL of the fluorescent sample dissolved in 45 μL of the test fluid (native human serum or the HEPES buffer control). Lipoplexes and PEGylated lipoplexes (containing labeled ONs) were the test samples. Free labeled ONs and the test fluid alone were used as controls. The fluorescence intensity fluctuations were recorded using SymPhoTime (Picoquant, Berlin, Germany) for at least 60 s, using a 488 nm laser. The resulting autocorrelation curves [31,32] were then fitted by a triplet-state dual-species model (Equation (1)) with SymPhoTime software:

(1) $$G\left(t\right)=\frac{1}{Ν\left(1-T\right)}\times \left[1-T+T\times \exp\left(\frac{-t}{\tau_{t}}\right)\right]\times \left[\frac{\gamma }{\left(1+\frac{t}{\tau_{t1}}\right)\sqrt{1+{\left(\frac{\omega_{0}}{z_{0}}\right)}^{2}\times \left(\frac{t}{\tau_{t1}}\right)}}+\frac{1-\gamma }{\left(1+\frac{t}{\tau_{t2}}\right)\sqrt{1+{\left(\frac{\omega_{0}}{z_{0}}\right)}^{2}\times \left(\frac{t}{\tau_{t2}}\right)}}\right]$$

where 2$\omega_{0}$ and $2z_{0}$ represent the diameter and the height of the detection volume, respectively. *T* is the percentage of molecules in triplet state and $\tau_{t}$ is the triplet relaxation time (the time particles spend in the triplet state). N×γ and N×(1−γ) represent the molecules with diffusion time $\tau_{t1}$ and $\tau_{t2}$, respectively. Using the SymPhoTime software, the fraction corresponding to each of the diffusion populations (free ONs and complexed ONs) was calculated based on the fitted $\;\rho_{1}$ and $\;\rho_{2}\;$ values using Equation (2):

(2) $$\;\;\;\;\;\;\;Fraction\;\left(\%\right)=\frac{\rho_{1}}{\rho_{1}+\rho_{2}}\times 100\%$$

### 2.6. Evaluation of Liposomes’ Interaction with Bacteria by Epifluorescence Microscopy

To investigate liposomes’ interaction with different bacterial envelopes (Gram-negative and Gram-positive), labeled liposomes were incubated with bacteria and visualized with an epifluorescence microscope. Rh-PE-labeled PEGylated and non-PEGylated DOTAP–DOPE liposomes were prepared using the methodology described above.

*Escherichia coli* K12 MG1655, *Staphylococcus aureus* Mu50 ATCC 700699, *Acinetobacter baumannii* NCTC 13423, *Enterococcus faecium* CECT 410, *Klebsiella pneumoniae* ATCC 11296 and *Pseudomonas aeruginosa* PAO1 were selected due to their relevance in blood infections and multiresistance. Bacteria were grown overnight in TSB broth at 37 °C and diluted to an OD_600_ of 0.680 in HEPES buffer (pH 7.4). The bacterial suspension was mixed with Rh-PE liposomes (with or without PEG), resulting in a concentration of 50 µM of the total lipids in the mixture and a bacterial concentration (OD_600_) of 0.6. After 1 h incubation with shaking (180 rpm) at 37 °C, the suspension was diluted in filtered distilled water to a final OD_600_ of 0.1. Smears were prepared on microscope glass slides (20 µL per slide well) by drying at 37 °C. The bacteria were observed on a Nikon Eclipse Ti SR epifluorescence microscope (Nikon, Tokyo, Japan), equipped with a QImaging Retiga R1 monochromatic camera (Burnaby BC, Canada) and a Nikon Plan-Apo 100 × 1.45 N.A. oil immersion objective lens (Nikon, Tokyo, Japan). Rh fluorescence was visualized using a G-2A longpass filter (excitation: 535 nm; emission: 580 nm) and maintaining the exposure time and the excitation intensity among the different samples; ten pictures of each sample were taken randomly, covering all the areas of the sample. The images were processed with NIS-Elements Advanced Research (Nikon, Tokyo, Japan).

### 2.7. Quantification of Liposomes’ Fusion with Different Bacterial Envelopes Using Lipid-Mixing Assay

To further understand the extent of interaction between liposomes and the different bacterial envelopes, a lipid-mixing assay based on fluorescence resonance energy transfer (FRET) was performed to quantify fusion [33].

Briefly, *E. coli*, *S. aureus*, *A. baumannii*, *E. faecium*, *K. pneumoniae* and *P. aeruginosa* were grown to their middle exponential phase, followed by a resuspension in previously heated (37 °C) 20 mM HEPES buffer (pH 7.4), resulting in a final OD_600_ of 0.680. The bacterial suspension was incubated at 37 °C with Rh-PE/NBD-PE liposomes (with or without PEG) at a final total lipid concentration of 50 µM and bacterial OD_600_ of 0.6. Every 10 min, 100 µL of each mixture was mixed in 100 µL of HEPES buffer and distributed into a 96-well plate. The fluorescence intensity of Rh was measured in a FLUOstar Omega Microplate Reader (BMG Labtech, Offenburg, Germany) at 590 nm under a steady-state excitation of 470 nm. After 60 min incubation, Triton X-100 detergent (0.2% (*v*/*v*) was added to the mixture, in order to completely disrupt the liposomes and lead to a maximal lipid probe dilution, and the final rhodamine fluorescence intensity was measured.

The obtained percentage of fusion was calculated using the following Equation (3) [34]:

(3) $$\%\;Fusion=\frac{F_{t}-F_{0}}{F_{max}-F_{0}}\;\times \;100$$

where *F_t_* is the fluorescence intensity of Rh at each time point and *F*_0_ and *F_max_* are its initial and final fluorescence intensity, respectively. The final fluorescence intensity was achieved after the addition of Triton X-100 detergent.

### 2.8. Assessment of NAM-ONs Internalization by Confocal Microscopy

The ability of the PEGylated liposomes to internalize NAM-ONs in Gram-positive and Gram-negative bacteria was studied using fluorescence in situ hybridization (FISH) followed by bacterial membrane staining.

*E. coli* K12 and *S. aureus* Mu50 were grown overnight in TSB broth at 37 °C followed by a dilution to nearly 1.6 × 10^6^ cells/mL and grown until the mid-exponential phase at 37 °C. FISH was then applied. Traditional in vitro FISH starts with a permeabilization/fixation step, needed to render the bacterial envelope permeable to the ONs, based on pretreating the bacteria with chemicals that are toxic for in vivo application [35,36,37]. In this study, FISH was applied without this pretreatment, based on Santos et al. with slight modifications [19]. The bacterial suspension containing 1.6 × 10^6^ cells/mL was centrifuged for 15 min at 8600× *g* and resuspended in HEPES buffer containing PEGylated lipoplexes (at a final concentration in the mixture of 400 nM HiLyte 488 labeled ONs). The mixture was incubated for 1 h, at 37 °C, and centrifuged for 10 min at 8600× *g*. The bacterial cells were washed in 500 μL of washing solution (0.005 M Tris base, 0.015 M NaCl, 0.1% (*v*/*v*) Triton-X, pH 10) for 15 min, at 37 °C, and centrifuged again (8600× *g*, 5 min). The bacteria were resuspended in 1% (*v*/*v*) DiD membrane dye in ethanol and incubated for 5 min at 37 °C. After centrifugation (8600× *g*, 5 min), bacterial cells were resuspended in sterile distilled water, and 20 μL of each sample was placed on a glass slide well and dried at 37 °C for microscopy visualization.

Microscope images were taken using a C1si laser scanning confocal microscope (CLSM) (C1si, Nikon Tokyo, Japan) and a 100x oil immersion objective (Plan Apo VC 100× 1.4 NA, Nikon, Tokyo, Japan). HiLyte 488 and DiD were excited by a 488 and 640 nm diode laser (CVI Melles Griot, Albuquerque, NM, USA). At least 10 images were acquired with the NIS-Elements AR software (Nikon, Tokyo, Japan) using an EMCCD camera (iXon Ultra 897, Andor Technology, CT, USA).

### 2.9. Determination of Cellular Localization of NAM-ONs by Bacterial Fractionation

To confirm the cellular localization of the labeled NAM-ONs in *E. coli* and *S. aureus*, after their contact with the lipoplexes, the bacterial cells were fractioned into a membrane fraction and a cytosol fraction, and the HiLyte 488 fluorescence intensity was measured in a FLUOstar Omega Microplate Reader (Offenburg, Germany). Overnight inocula of *E. coli* K12 and *S. aureus* Mu50 were diluted in HEPES buffer to an OD_600_ of 0.1, in the presence of PEGylated lipoplexes for 1 h, at 37 °C. The amount of lipoplexes added was such that 400 nM of labeled ONs were present in the final bacteria–lipoplexes mixture. Thereafter, a fractionation protocol adapted from Bandula et al. was followed [38]. In brief, bacteria were centrifuged (3000× *g*, 20 min), resuspended in 10 mM Tris–150 mM NaCl (pH 7.4) and washed with 50 mM Tris (pH 7.6) to remove lipoplexes that did not interact with bacteria. To obtain the fraction associated with the outer membrane (membrane fraction), bacteria were centrifuged (3000× *g*, 20 min) and resuspended in 50 mM Tris buffer solution containing 0.05% (*v*/*v*) Triton X-100 (pH 7.6), for 1 h, at room temperature (RT). After a new centrifugation (same conditions), the fluorescence intensity of the supernatant (membrane fraction) was measured with the fluorometer. To obtain the fraction associated with the cytosol, bacteria were centrifuged, and the pellet was resuspended in a more astringent 50 mM Tris buffer containing 1% (*v*/*v*) Triton X-100 (pH 7.6), for 1 h, at RT. After the last centrifugation (3000× *g*, 20 min), the fluorescence of the supernatant (cytosol fraction) was measured. The percentage (%) of ONs in each fraction was calculated using the following Equation (4):

(4) $$\%\;ONs{\;}_{fraction}=\frac{F_{fration}}{F_{cytosol}+F_{membrane}}\times 100$$

where $F_{fraction}$ represents the fluorescence intensity of the bacterial fraction; $F_{cytosol}\;and\;F_{membrane\;}$ represent the fluorescence intensity of the cytosol and membrane fractions, respectively. All these fluorescence values were previously corrected for autofluorescence (by subtracting the value obtained in each step for the bacterial autofluorescence).

As a control, the same fractionation protocol was applied to bacteria stained only with DAPI (a cell-permeable dye known to stain the cytosol by binding to DNA).

### 2.10. Statistical Analysis

Experiments were performed in triplicates on independent days. Significance between the means of the experimental groups was evaluated using two-way analysis of variance (ANOVA), applying Sidak’s multiple-comparison test. The software GraphPad Prism 7 was used and a *p*-value of ≤ 0.05 was considered as significant (*p* ≤ 0.05, *; *p* ≤ 0.01, **; *p* ≤ 0.001, ***; *p* ≤ 0.0001, ****).

## 3. Results and Discussion

### 3.1. Characterization of the Liposomes and Lipoplexes

Cationic liposomes, constituted of DOTAP and DOPE in a 1:1 mol ratio, were prepared following the lipid hydration method [19,39]. Assessment of their hydrodynamic size and zeta potential was performed to ensure the reproducibility of the results for the following experiments. The liposomes had an average hydrodynamic diameter of 91 ± 9 nm, with a polydispersity (PDI) of 0.25 ± 0.05 nm. The average zeta potential was +49 ± 4 mV. After complexation with NAM-ONs, the zeta potential, hydrodynamic diameter and PDI of the liposomes did not change significantly. Differently, upon PEGylation of the lipoplexes, the final zeta potential of the lipoplexes decreased to +14 ± 3 mV and the average hydrodynamic diameter increased to 112 ± 10 nm, while the PDI did not vary significantly. These results are in accordance with previous studies and confirm the success of PEG insertion [19,40].

Rh/NBD-labeled liposomes, prepared for the interaction studies, had an identical average zeta potential as their unlabeled counterparts and a hydrodynamic diameter of 86–106 nm. The final zeta potential of PEGylated labeled liposomes was around +16–18 mV and the average hydrodynamic diameter was 93–113 nm.

### 3.2. Complexation Stability of the Lipoplexes in Human Serum

Oftentimes, the stability of formulations in biologically relevant fluids is disregarded in biochemical and microbiology studies, causing premature failure of novel antimicrobial strategies. In order to treat bloodstream infections, commonly caused by multidrug-resistant bacteria, human serum needs to be considered as the first delivery barrier. Upon intravenous administration, lipoplexes will come into contact with blood, and interactions with serum components may result in premature release of the NAM-ONs from the lipoplexes [41]. As this is to be avoided, the stability of liposomes–ONs association was studied, over time, in undiluted human serum using FCS [30,42].

**Figure 2:**
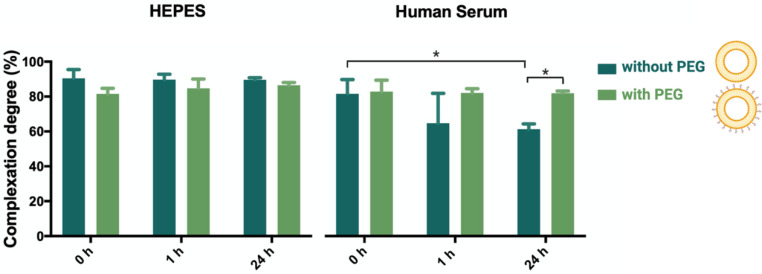
Percentage of association degree between liposomes and ONs in lipoplexes (without PEG) and PEGylated lipoplexes (with PEG), determined by single-color FCS analysis in HEPES buffer and in undiluted human serum at 0, 1 and 24 h incubation, at 37 °C. Three independent experiments were done. Results are represented as mean values and respective standard deviations. Statistical differences are indicated when appropriate (*p* ≤ 0.05, *).

Figure 2 shows a high degree of association between NAM-ONs and liposomes in HEPES buffer (over 24 h), with more than 80% of the NAM-ONs complexed to the liposomes, with and without PEG. Differently, after 24 h incubation in human serum, the same high level of NAM-ONs association (81%) was only achieved for PEGylated lipoplexes, while it was significantly lower for non-PEGylated lipoplexes (61%). Fast release of siRNA in serum was observed before from non-PEGylated DOTAP–DOPE liposomes [42]. Differently, in liposomes also containing 10% (*v*/*v*) DSPE-PEG, more than 90% release of siRNA was reported after 1 h incubation in human serum by Dakwar et al. [42]. However, it should be noted that in that study the pre-PEGylation was performed by including DSPE-PEG already in the initial lipid mixture before vesicle formation. Instead, in the present work, post-insertion of PEG chains, i.e., after vesicle formation, was used. This is the first time that the release of ONs from post-PEGylated lipoplexes has been studied, and our results suggest that post-PEGylation provides a shielding effect, preventing the release of ONs in serum. This, together with the already proven colloidal stability of the used liposomes in blood [43], is a promising result for a successful in vivo administration. Nonetheless, future work could also consider the effect of the circulatory flow rate to mimic in vivo hydrodynamics before validation.

### 3.3. Interaction and Fusion of Liposomes with Different Bacterial Envelopes

**Figure 3:**
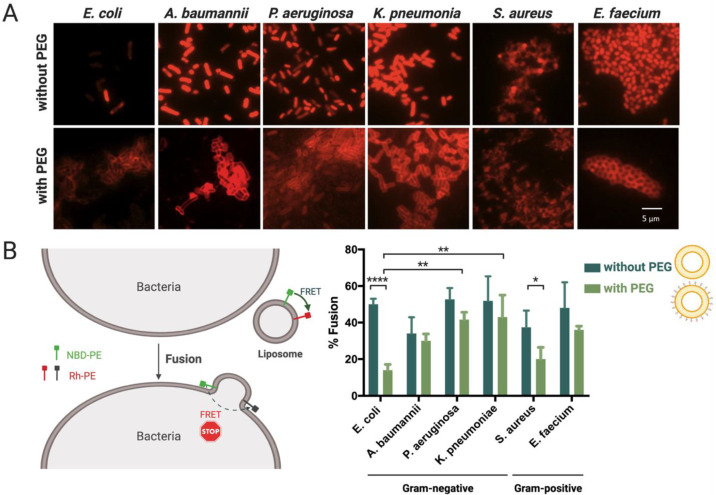
Interaction and fusion of liposomes, with and without DSPE-PEG, with clinically relevant bacteria. (**A**) Representative epifluorescence microscopy pictures of bacteria incubated with Rh-labeled liposomes. Scale bar represents 5 μm. (**B**) Percentage of Rh/NBD liposomes fused with bacteria. Three independent experiments were done. Results are represented as mean values and respective standard deviations. Statistical differences are indicated when appropriate (*p* ≤ 0.0001, ****; *p* ≤ 0.01, **; and *p* ≤ 0.05, *). Figure 3B (**left**) was created with BioRender.com. (Accessed on 1 June 2021).

After proving that the liposomes are able to stably transport NAM-ONs in human serum, we aimed to investigate liposomes’ capability to interact and fuse with different bacterial envelopes, as this is required for intracellular delivery of ONs in bacteria, and the effect of PEG in such interaction. Different clinically relevant bacteria responsible for bloodstream infections were used: *Escherichia coli* K12, *Staphylococcus aureus* Mu50, *Acinetobacter baumannii*, *Enterococcus faecium*, *Klebsiella pneumoniae* and *Pseudomonas aeruginosa*, and incubated with empty liposomes, with and without PEG. The interaction between bacteria and empty liposomes was analyzed using epifluorescence microscopy (Figure 3A), and their fusion with bacteria was quantified by a lipid-mixing assay (Figure 3B).

Figure 3A shows microscopy images of the different analyzed bacteria after contact with either Rh-labeled liposomes or Rh-labeled PEGylated liposomes. Both types of liposomes interacted with all bacteria tested. However, the association pattern appeared different, as a homogeneous staining of cells was seen in the case of non-PEGylated liposomes, while a halo surrounding the cells was seen for the PEGylated ones. This points to the fact that, in the absence of PEG, liposomes interact strongly with bacterial membranes, which is in accordance with previous results [44,45]. Additionally, liposomes seem to interact with both Gram-negative and Gram-positive bacteria. Adsorption of cationic liposomes on the bacterial surface may be explained by electrostatic interactions with LPS in Gram-negative bacteria and teichoic acids in Gram-positive bacteria [11].

We investigated how the observed interaction translated into fusion between the liposomes (presumably triggered by the DOPE lipid) and the bacterial membranes, using a FRET-based lipid mixing assay [46,47,48]. This technique is based on the transference of energy between two headgroup-labeled phospholipids, Rh-PE (acceptor) and NBD-PE (donor), present at the same concentration in liposomes. Fusion of such a dual-labeled liposome with a bacterial cell increases the distance between the two fluorophores, therefore interrupting the energy transfer and decreasing the measured fluorescence intensity of the acceptor (rhodamine) (Figure 3B).

From Figure 3B, it is noticeable that the interaction observed in Figure 3A leads to fusion in all tested bacteria. Moreover, the fusion results do not show a direct correlation between fusion efficiency and the nature of the bacterial envelope (Gram-negative vs. Gram-positive bacteria). In our study, it is apparent, from the lack of differences between Gram-negative and Gram-positive bacteria, that the presence of an outer membrane (OM) is not necessarily essential for fusion, as would be expected. It rather seems that the liposomes can destabilize the peptidoglycan of Gram-positive bacteria and fuse with their inner membrane (IM). Several studies also demonstrated that fusogenic liposomes are able to pass through the cell wall of different Gram-positive bacteria and deliver antibiotics [49,50,51,52]. Scriboni et al. tested different vancomycin liposomal formulations against *S. aureus* and found that the fusogenic liposomes were the most successful in inhibiting bacterial growth. They also found that free vancomycin had a better inhibitory effect in the early stages of biofilm formation than the liposomal formulation, but the reverse happened in a mature biofilm, once the peptidoglycan layers became thicker. They postulated that the liposomes had an increased ability to penetrate the peptidoglycan layers, whereas the free vancomycin remained trapped in the cell wall [49]. Concerning the different levels of fusion observed in Figure 3 among bacteria from the same Gram-type, similar results were obtained by Wang and his colleagues, suggesting that different levels of phosphatidylethanolamine (PE) and phosphatidylglycerol (PG) on the bacterial envelope may affect different levels of fusion [48].

The results presented in Figure 3B also show an overall lower fusion of post-PEGylated liposomes with all bacteria tested, although the differences are only statistically significant in *E. coli* and *S. aureus* (*p* ≤ 0.0001 and *p* ≤ 0.05, respectively). While PEGylation of lipoplexes improves their stability in biofluids, it has, however, been reported that PEGylation also lowers the interaction of liposomes with cells by limiting the electrostatic and fusogenic interaction of the cationic liposomes with the negatively charged membranes [53,54,55]. Nonetheless, our results for post-PEGylated liposomes show they still have the capacity to fuse with bacterial membranes. The fact that PEG is added after the formation of the liposomes may explain why they are still able to interact with bacteria [56]. Interestingly, the Gram-negative *E. coli* and the Gram-positive *S. aureus* were the bacteria with lower percentages of fusion with PEGylated liposomes, which is also denoted by a lower rhodamine fluorescence intensity in Figure 3A, compared to the other bacteria.

A control using liposomes was performed to evaluate the extent of self-fusion events. Liposomes, with and without PEG, were tested with both fluorophores against their unlabeled counterparts (Figure S1). The results show that the percentage of fusion between liposomes is significantly lower than the observed fusion with bacteria (*p* > 0.001). The only exception is related to the fusion of PEGylated liposomes with *S. aureus* and *E. coli*, which was very residual (*p* > 0.05). These results indicate that the events of self-fusion do not significantly promote the decrease in FRET that was observed when fusion of liposomes with bacteria was investigated. Additionally, fluorescence did not vary significantly over time when rhodamine emission was monitored under rhodamine excitation (530 nm), instead of NBD (470 nm), indicating that the decrease in FRET was not promoted by photobleaching (not shown).

### 3.4. Characterization of NAM-ONs Internalization and Cellular Localization

We aimed to investigate how much of the observed interaction and fusion is needed to successfully deliver NAM-ONs into bacteria. Therefore, we used *E. coli* and *S. aureus* to test if the significantly lower interaction with post-PEGylated liposomes negatively affects intracellular delivery of NAM-ONs, as PEG appears to be pivotal in future in vivo applications. Using *E. coli* and *S. aureus* as model Gram-negative and Gram-positive bacteria, respectively, we investigated (i) the intracellular delivery of NAM-ONs using confocal microscopy and (ii) their relative distribution within the cytosol and membrane by bacterial cell fractionation.

**Figure 4:**
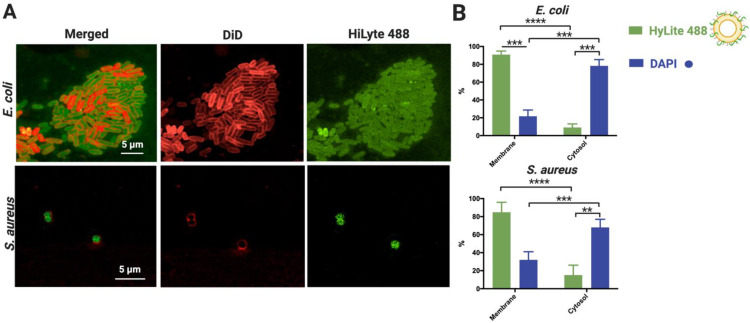
Assessment of HiLyte 488 labeled NAM-ONs internalization by post-PEGylated lipoplexes in HEPES buffer, upon 1 h incubation. (**A**) Representative CLSM images of *E. coli* (**top**) and *S. aureus* (**bottom**) showing NAM-ONs (green fluorescence) internalized in the cytosol. Bacterial membranes were labeled with DiD dye (red fluorescence). Merged and separate channels are represented. Scale bar represents 5 μm. (**B**) Percentage (%) of NAM-ONs localized in the cytosol and in the membrane, calculated using Equation (4). A DAPI control of cytosol labeling was performed. Three independent experiments were done. Results are represented as mean values and respective standard deviations. Statistical differences are indicated when appropriate (*p* ≤ 0.0001, ****; *p* ≤ 0.001, ***; and *p* ≤ 0.01, **).

Visualization of the ONs internalization was obtained after performing FISH with the post-PEGylated lipoplexes (carrying HiLyte 488 ONs) followed by membrane staining with DiD. As presented in Figure 4A, the ONs can be seen not only at the edge of the cells but also inside both *E. coli* and *S. aureus*. This observation proves that the post-PEGylated lipoplexes are at least partially able to release ONs in both Gram-negative and Gram-positive bacteria, mediated by liposomal fusion with the envelope.

To quantify the relative amount of ONs delivered in the bacterial cytosol, we performed bacterial cell fractionation after contact with the post-PEGylated lipoplexes (Figure 4B). We found that 9.1% and 15.1% of the ONs carried by the liposomes were present in the cytoplasm of *E. coli* and *S. aureus*, respectively, with the remaining amount colocalized with the membrane. As expected, the DAPI control was mostly found in the cytosolic fraction (68–78%), validating the obtained fractionation results. The small DAPI fraction associated with the membrane is in agreement with previous reports [57].

Overall, the results show that fusion of lipoplexes does not necessarily lead to the delivery of all the transported cargo. Moreover, despite the apparent low levels of labeled ONs in the cytosol of *E. coli* and *S. aureus* (Figure 3B), post-PEGylated liposomes deliver ONs inside both bacteria to efficiently hybridize with rRNA and label the bacteria (Figure 4A).

Whether such amount of delivered NAM-ONs is sufficient for future successful antisense therapy is unknown, as there are no studies showing the amount of internalized ASOs that is needed to efficiently inhibit bacterial gene translation. Nonetheless, the fact that the number of copies of rRNA in a bacterium is significantly higher than mRNA is promising [58].

After decades of study, the therapeutic potential of ONs remains largely unrevealed. Attempts to fundamentally explain the biological activity and the biophysical properties of nucleic acid loaded carriers are scarce but greatly needed to allow the design of better delivery systems for nucleic acids. DOTAP–DOPE liposomes have been previously shown to efficiently deliver ONs into eukaryotic cells [42,59,60] and H. pylori [19]. However, this is the first study on the interaction of lipoplexes with different bacteria (i.e., different cell wall characteristics) that are clinically relevant and on their potential for intravenous application. The formation of the lipoplexes is a nonlaborious process that sandwiches the ONs into lipid multilayers and was therefore chosen for this study [59]. Some of the ONs are expected to stay on the surface after the complexation, and post-PEGylation was used to prevent their premature release after contact with human serum [60]. Although some questions regarding the mechanism of fusion of liposomes with Gram-positive bacteria still need to be addressed, this strategy proved to be efficient towards the successful delivery of ONs into both bacteria, without risking their premature release from the liposomes in vivo, as this formulation remained stable in serum for at least 24 h.

## 4. Conclusions

The results presented in this study are promising as they indicate that post-PEGylated DOTAP–DOPE liposomes successfully fuse with both Gram-negative and Gram-positive bacteria. Additionally, the formulation may be considered to treat bloodstream infections as it remains stable in human serum. Therefore, ONs that are designed to hybridize with essential genes or genes associated with antibiotic resistance can potentially be delivered into different clinically relevant bacteria. Future research will focus on dose-dependent assays using ONs to evaluate their antimicrobial efficiency and test their in vivo performance.

## Data Availability

The data presented in this study are available in the article.

## Supplementary Materials

The following are available online at https://www.mdpi.com/article/10.3390/pharmaceutics13070989/s1, Figure S1. A self-fusion control with PEGylated and non-PEGylated liposomes was performed (“liposomes” bars), by mixing labeled and unlabeled liposomes on the same category (PEGylated and non-PEGylated). Three independent experiments were done and the results were compared with fusion with the bacteria. Not significant (n.s.).
