# Supplementary material for: Lipoplexes to Deliver Oligonucleotides in Gram-Positive and Gram-Negative Bacteria: Towards Treatment of Blood Infections
Source: Pharmaceutics. 2021 Jun 29;13(7):989. doi: 10.3390/pharmaceutics13070989 (PMC8309032; doi:10.3390/pharmaceutics13070989)
Supplement: Supplementary file 1 [file pharmaceutics-13-00989-s001.zip › pharmaceutics-1264378-supplementary.pdf]

pharmaceutics

MDPI

Supplementary Materials: Lipoplexes to Deliver Oligonucleotides in Gram-Positive and Gram-Negative Bacteria: Towards Treatment of Blood Infections

Sara Pereira, Rita Sobral Santos, Luís Moreira, Nuno Guimarães, Mariana Gomes, Heyang Zhang, Katrien Remaut, Kevin Breackmans, Stefaan De Smedt and Nuno Filipe Azevedo

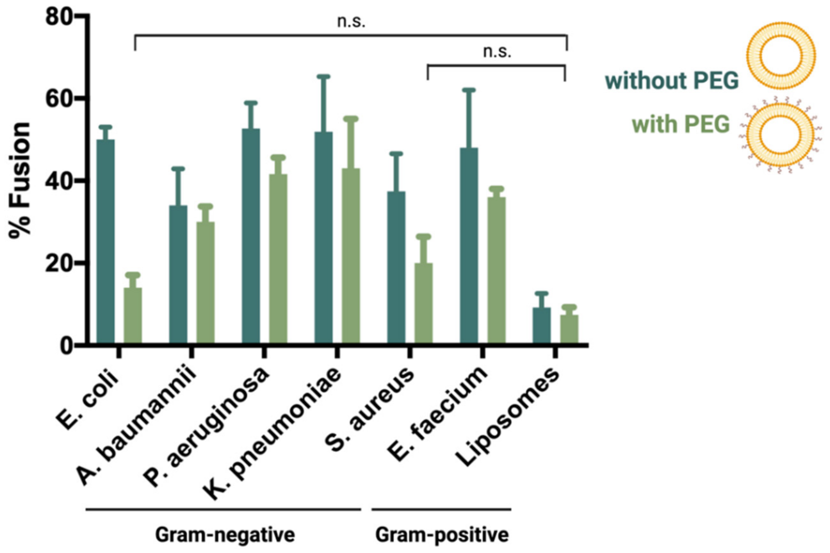

Figure S1. A self-fusion control with PEGylated and non-PEGylated liposomes was performed ("liposomes" bars), by mixing labeled and non-labeled liposomes on the same category (PEGylated and non-PEGylated). Three independent experiments were done and the results were compared with fusion with the bacteria. Not significant (n.s.).
